# MAPK-ERK Pathway

**DOI:** 10.3390/ijms24119666

**Published:** 2023-06-02

**Authors:** Jong-In Park

**Affiliations:** Department of Biochemistry, Medical College of Wisconsin, Milwaukee, WI 53226, USA; jipark@mcw.edu

The name extracellular signal-regulated kinase (ERK) was first used for a cell cycle regulating Ser/Thr protein kinase cloned in mammalian cells [1], which was termed ERK1 and also mitogen-activated protein kinase (MAPK) in another study [2]. It is now widely known that ERK is the effector kinase in a highly specific three-layered kinase cascade with pivotal roles in various cellular processes, including cell cycle progression, survival, and differentiation. This pathway’s deregulation is implicated in various pathophysiological conditions. Recent studies have substantially expanded our knowledge of the MAPK-ERK pathway signaling, allowing the development of diverse potential therapeutic strategies targeting the pathway. This Special Issue covers recent advances in the molecular mechanisms and functions of MAPK-ERK signaling in different biological contexts.

As the critical focal point of the Raf/MEK/ERK pathway signaling, ERK1 and ERK2 (referred to as ERK1/2) have essential roles throughout development and adulthood. Capolongo et al. introduce different roles of ERK1/2 in embryonic kidney development, focusing on renal morphogenesis and differentiation, and several renal diseases, including polycystic kidney disease, glomerulonephritis, diabetic nephropathy, and unilateral ureteral obstruction [3]. They review the literature investigating the effects of ERK1/2 signaling in the renal tubule in mature kidneys and discuss the molecular mechanisms by which ERK1/2 regulates the function of several ion channels and transporters implicated in acid–base and electrolyte homeostasis in the nephron. The most notable mechanisms include ERK1/2-mediated transcriptional and post-transcriptional regulation of several nephron channels and transporters in the organ.

Albert-Gascó et al. review the effects of ERK1/2 signaling on developing social and emotional behaviors, focusing on the molecular mechanisms that the kinases mediate in the amygdala and amygdala-related areas [4]. The authors first provide an overview of ERK1/2 signaling in neurons and its molecular mechanisms identified in different subcellular locations spanning the cell surface to the nucleus. They then discuss the role of ERK1/2 in configuring the emotional-related anatomical areas and processing emotional and social information, spanning emotional brain development, late embryonic and early postnatal development, and adult emotional and memory systems. They also review the role of ERK1/2 in diverse adult systems, including spatial memory, social behavior, and fear, suggesting that the ubiquity of ERK1/2 signaling in the central nervous system may indicate the significance of these kinases in multiple cognitive processes. This review also focuses on the implication of ERK1/2 dysfunction in neurodegenerative diseases, including Parkinson’s disease, Alzheimer’s disease, amyotrophic lateral sclerosis, Huntington’s disease, and prion diseases, as well as autism spectrum disorders. The authors provide a comprehensive review and updates on recent advances in ERK1/2 signaling in neurons.

Epithelial-mesenchymal transition (EMT) describes the trans-differentiation of epithelial cells into a mesenchymal phenotype. EMT is necessary for the normal development of higher eukaryotes and is involved in certain pathophysiological conditions. For example, different oncogenic signaling pathways and tumor microenvironment signals often trigger EMT to promote tumorigenesis. Olea-Flores et al. review three significant mechanisms of EMT, with a focus on the type 3 EMT associated with cancer metastasis and progression [5]. In this review, the authors provide a comprehensive overview of different typical and atypical members in the ERK family concerning their unique structural and functional features and their functional Relationship with EMT in various tumors, including cancers of the lung, breast, colon, cervix, ovary, and prostate.

Although mainly known as cell-proliferative kinases, ERK1/2 can mediate cell cycle arrest and death. For example, ERK1/2 activation, in response to oncogenic RTK, Ras, or Raf mutants, can induce growth inhibition in normal cells and specific tumor cells. Wu et al. review recent updates on the molecular mechanisms underlying the Raf/MEK/ERK pathway-mediated growth inhibitory signaling, focusing on the mechanisms that determine the magnitude of pathway activity, spatial-temporal regulation, and non-canonical functions of the molecular switches in this pathway [6]. The growth inhibitory signaling of ERK1/2 might indicate that cells must constrain these kinases’ activity within a desired signal intensity range to achieve proper growth and proliferation. As such, cells would interpret their too-low and too-high signal intensity as a signal to trigger anti-proliferative/survival responses. This concept is a relatively less-known aspect of ERK1/2 signaling.

Viral pathogenesis requires the manipulation of signaling pathways in host cells. Notably, DNA viruses manipulate ERK1/2 signaling in cells to promote viral internalization, dysregulate the cell cycle, regulate viral replication through the host-cell machinery, and prevent host-cell death. Thus, elucidating how DNA viruses regulate the ERK1/2 pathway is critical for a better understanding of viral pathogenesis. DuShane and Maginnis review the dynamic nature of ERK1/2 signaling and its importance in regulating cellular fates that influence viral infection [7]. Interestingly, many DNA viruses converge at the utilization of the ERK1/2 pathway during infection despite the disparity in tissue tropism and disease manifestations. In a separate research article in this topic series, these authors also identify the role of ERK1/2 in promoting the infection of JC polyomavirus, a ubiquitous human pathogen, by activating downstream transcription factors [8]. Their data strongly suggest that the ERK1/2 pathway is a potential therapeutic target for viral infection.

This topic series also accommodates several research articles that suggest novel ERK pathway effects in different biological contexts. First, the hepatocyte growth factor exerts cardioprotective effects against the cardiotoxicity caused by the chemotherapeutic agent doxorubicin. Gallo et al. explore the molecular mechanism underlying this beneficial effect in the H9c2 cell culture model and demonstrate the role of ERK1/2 signaling in mediating the growth factor’s anti-genotoxic and pro-survival effects [9]. Second, fetal and hypertrophic remodeling are hallmarks of cardiac restructuring for heart failure. Kubin et al. hypothesized that ERK1/2 signaling is a key factor regulating the development or prevention of heart failure [10]. Using the models of cultured adult rat cardiomyocytes and myocytes in the failing human myocardium, they test this hypothesis and support the significance of ERK1/2. Third, Activin B is a transforming growth factor superfamily member commonly expressed in various tissues. Using an anagen induction assay and an in vitro vibrissae culture model, Tang et al. demonstrate that Activin B may promote mouse vibrissae growth by stimulating hair matrix cell proliferation and cell cycle progression through ERK1/2 signaling [11]. Fourth, hypoxia-inducible factor 1 (HIF-1) plays a pivotal role in tumor adaptation to micro-environmental hypoxia, and it also exerts essential roles in angiogenesis and tumor development. Gong et al. show that vanillic acid, a dietary phenolic compound reported to exhibit anticancer properties, inhibited HIF-1a protein synthesis in various human cancer cell lines by suppressing the Raf/MEK/ERK and mammalian target of rapamycin pathways simultaneously [12]. Lastly, ERK3 is an atypical member of the MAPK family that harbors a kinase domain in the N-terminus and a long C-terminus extension. Little is known about the regulation of its enzymatic activity and cellular functions. Elkhadragy et al. investigated the role of the elongated C-terminus extension in regulating ERK3 kinase activity and its ability to promote cancer cell migration and invasion [13]. They show that the deletion of the C-terminus tail decreases the kinase activity of ERK3 and abolishes the ability of ERK3 to promote septin 7-mediated lung cancer cell migration, providing novel insights into the molecular mechanism underlying ERK3 regulation. It is hoped that these novel observations will help in the ongoing efforts to understand better the role of the ERK pathways in different biological contexts.
